# Isolation and Molecular Identification of Lactic Acid Bacteria Using 16s rRNA Genes from Fermented* Teff* (*Eragrostis tef* (Zucc.)) Dough

**DOI:** 10.1155/2018/8510620

**Published:** 2018-08-05

**Authors:** Belay Tilahun, Anteneh Tesfaye, Diriba Muleta, Andualem Bahiru, Zewdu Terefework, Gary Wessel

**Affiliations:** ^1^Department of Biotechnology, College of Natural Sciences, Wolkite University, P.O. Box 07, Wolkite, Ethiopia; ^2^Institute of Biotechnology, Addis Ababa University, Addis Ababa, Ethiopia; ^3^Addis Ababa Institute of Technology, Addis Ababa University, Addis Ababa, Ethiopia; ^4^MRC-ET Molecular Diagnostics Laboratory, Addis Ababa, Ethiopia; ^5^Department of Molecular Biology, Cellular Biology and Biochemistry, Brown University, Providence, RI 02912, USA

## Abstract

Injera is soft fermented baked product, which is commonly prepared from teff (*Eragrostis tef *(Zucc.)) flour and believed to be consumed on daily basis by two-thirds of Ethiopians. As it is a product of naturally fermented dough, the course of fermentation is done by consortia of microorganisms. The study was aimed at isolating and identifying some dominant bacteria from fermenting* teff (Eragrostis tef)* dough. A total of 97 dough samples were collected from households, microenterprises, and hotels with different fermentation stage from Addis Ababa. The bacterial isolates obtained from the fermenting* teff* dough samples were selected on the basis of their acid production potentials. A total of 24 purified bacterial isolates were found to be Gram-positive (they are coccus and rod under microscope) and were good acid producers. Genomic DNA of bacterial isolates were extracted using Invisorb® Spin DNA Extraction kit. 16S rRNA of bacterial isolates were amplified using the bacteria universal primers (rD1 and fD1). The amplified product was sequenced at Genewiz, USA. Sequence analysis and comparison with the resources at the database were conducted to identify the isolated microbes into species and strain levels. The bacterial isolates were identified as* Lactobacillus paracasei, Lactobacillus brevis, Enterococcus durans, Enterococcus hirae, Enterococcus avium, *and* Enterococcus faecium*. All identified lactic acid bacteria were able to produce acid at 12 h time of incubation. This study has confirmed the presence of different bacterial species in the fermenting* teff* dough and also supports the involvement of various groups of bacterial species in the course of the fermentation.

## 1. Introduction

A wide variety of fermented foods and beverages are consumed in Ethiopia being prepared from a wide range of raw materials using traditional techniques. These include* injera, kocho, tella, awaze, borde, *and* tejj* [1].* Injera *is one of the fermented foods that is made from different cereals, including sorghum,* teff*, corn, wheat, barley, or a combination of some of these cereals [2].* Injera* from* teff* (*Eragrostis tef*) is much more relished by most Ethiopians than that from any other source. It is a thin soft fermented baked food usually obtained after the flour of cereals has been subjected to 24 to 96 h of fermentation depending on the ambient temperature [1, 3].

The fermentation process uses natural inoculants from different sources in a mixed form [4].* Teff injera* is getting popularity in the developed world because of its gluten free nature and being a whole grain product [5].* Teff* is a cereal crop which is mainly cultivated in Ethiopia for the purpose of making* injera *[2, 3]. For injera making,* teff* grain is considered by many as superior when compared to other cereal grains used in the country [6].

Research activities that investigate the microbial diversity and their roles during the course of fermentation of locally fermented products including* injera* have been started in the early 1980s in Ethiopia [7]. However, researches on such regard are not recent and/or limited to only [4] investigating microbial ecology of* injera* employing only phenotypic characterization of some of the fermenting microbial flora [7, 8] and the use of phenotypic characterization of fermenting microbial flora of fermenting dough during the preparation of* injera* employing only biochemical identification [9]. Only a very limited research activity was conducted recently using the present state-of-the-art technology for identifying of the fermenting microbial flora of local products [10, 11].

So, it is important to identify microorganisms with molecular approaches which have been developed to provide more rapid and accurate identification of bacteria using 16S rRNA gene sequences [12].

Therefore, the study was aimed at conducting molecular identification of microorganisms found in fermented* teff* dough hoping to give better understanding of microbial community found in fermented* teff *dough. The general objective of the study was to isolate, identify, and characterize lactic acid bacteria from fermented* teff *(*Eragrostis tef*) dough.

## 2. Materials and Methods

### 2.1. Isolates Designation

Microbial isolates were designated as follows: AAUBT for bacterial isolates followed by numbers and capital letters A, B, and C which represent the range of time of fermentation. A represents 48 h, B represents 60 h, and C represents 72 h of fermentation.

### 2.2. Sample Collection and Description of the Study Sites

A total of 97 teff dough samples (two hundred grams each) were collected from each of 14 sampling sites of fermenting dough samples at 48 h, 60 h, and 72 h time of fermentation [12] from hotels, households, and* injera* baking microenterprises in Addis Ababa, the capital of Ethiopia located at latitude of 8°58′N, longitude of 38°47′E, and altitude/elevation of 2324 m (7625 ft.). The samples were transported aseptically to Holeta Biotechnology Institute, Microbial Biotechnology Laboratory, for processing and microbial isolation. Sample processing, laboratory isolation, and identification of bacteria were carried out at Holeta Biotechnology Institute, Microbial Biotechnology laboratory. Molecular characterization was carried out at MRC-ET Molecular Diagnostics Laboratory, Addis Ababa, Ethiopia, and DNA sequencing was done at Genewiz in collaboration with Brown University, Boston, USA.

### 2.3. Isolation and Selection of Bacteria

Ten-gram dough from each sample was transferred aseptically into separate flask with 90mL sterile 0.1% peptone water and homogenized. Aliquots of 0.1 mL from appropriate dilutions were spread plated on presolidified de Man, Rogosa, and Sharpe (MRS) agar and incubated at 30°C for 48-72 h. Representative 10-20 colonies of lactic acid bacteria were randomly picked from countable MRS agar plates. Each bacterial isolate was purified by repeated streak-plating on MRS agar for three times. The pure isolates were maintained on MRS agar slants at 4°C and subcultured every four weeks until required for characterization [1]. The isolates were further examined for cellular morphology and staining characteristics using Gram stain and acid production test [13]. Cell morphology and colonial characteristics were observed on MRS agar [14].

#### 2.3.1. Acid Production Test of Bacteria

Bacterial isolates were refreshed on MRS broth, and then the broth was incubated at 37°C for 24 h. Turbidity of bacterial suspension was adjusted to 0.1 to 0.5 McFarland standard using spectrophotometer at the absorbance of 600 nm [13]. Each well of ELIZA plates was filled with 990 *μ*l of MRS broth with Bromocresol Green (0.04 gm/1000 ml) with pH range of 3.8-5.4. The first row of wells of the ELIZA plates served as a negative control without being inoculated with bacterial isolates. A total of 149 isolates of lactic acid bacteria were inoculated separately into each well with duplication. Finally, the plates were incubated at 37°C for 12 to 48 h. The formation of yellow color on the well indicated a positive result for fermentation or acidification, whereas the absence of color change was considered as a negative result.

### 2.4. Molecular Characterization of Bacteria

#### 2.4.1. Genomic DNA Extraction

Bacterial isolates were subcultured on MRS medium and incubated at 30°C for 48-72 h; i.e., 30 were selected on the basis of their morphological and acid production characteristics. The DNA of LAB isolates were extracted and purified using an Invisorb Spin DNA Extraction kit, according to the instructions of the manufacturer [15].

#### 2.4.2. Amplification of 16S rDNA of Bacterial Isolates

Fragments of the 16S rRNA genes of each bacterial isolate were separately amplified using the eubacteria universal primers rD1 (5′-AGA GTT TGA TCC TGG CT C AG-3′) and fD1 (5′-AAG GAG GTG ATC CAG CC-3′) [16]. For amplification of 16S rDNA genes of each bacterial isolate, PCR reaction mixtures (50 *μ*l) contained 1*μ*l of the extracted DNA, 5 *μ*l dNTPs, 1 *μ*l of each of the primers rD1 and fD1, 1 *μ*l of* Taq *DNA polymerase (Fermentas, St. Leon-Rot, Germany), and 5 *μ*l PCR buffer. To this content reverse osmosis purified water up to volume of 50 *μ*l was added. The temperature program and the cycle of reactions were as initial denaturation step at 95°C for 60 sec, followed by 35 cycles of denaturation at 94°C for 60 sec, primer annealing at 51°C for 30 sec, and primer extension at 72°C for 60 sec with a final extension at 72°C for 60 sec [17]. After running the PCR, the amplicons of LABs were separated by gel electrophoresis using 3% Agarose gel and 1 *μ*L loading dye with 5 *μ*L PCR products and stained with ethidium bromide for gel documentation.

### 2.5. Cloning and Sequencing of 16S rDNA Bacteria

**Figure 1:**
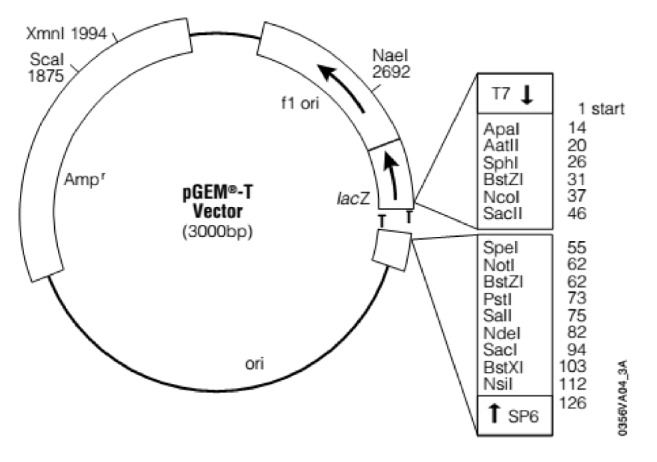
pGEM-T or pGEM-T easy vector (Genewiz, 2018).

The PCR amplified genomic region of interest (i.e., 16S rDNA) of each isolate was ligated into pGEM®-T easy vector (Figure 1) according to manufacturer's instructions, transformed into XL1-blue cells, and inoculated into medium containing 100*μ*g/ml ampicillin for selection. Isolates were grown for 16 h at 37°C with vigorous shaking. Plasmids were isolated as described in QIA quick Gel Extraction Kit. DNA was then sequenced by automated DNA sequencer (ABI model 377; Applied Biosystems) at Genewiz.

### 2.6. Phylogenetic Analysis

DNA sequences were edited, and consensus sequences were obtained using the Bioedit software package. Final sequences were then aligned using CLUSTAL (version: 1.2.4) [18] for each of the sequences. The sequences of bacterial isolates of this study were then compared to those in GenBank (National Centre for Biotechnology Information; http://www.ncbi.nih.gov/) using the Basic Local Alignment Search Tool [19] for nucleotide sequences (*blastn*). Phylogenetic tree construction was performed using the Maximum Likelihood method based on the Tamura-Nei model with MEGA 6.06 [20, 21].

## 3. Results and Discussion

### 3.1. Isolation of Bacterial Isolates

From 97 samples collected and processed a total of 249 bacterial isolates were recovered and purified. Purified isolates that are found to be Gram-positive (they are coccus and rod under microscope, Table 1) were tested for the bacterial acid production potential.

### 3.2. Acidification Test for Bacterial Isolates

Out of 249 bacterial isolates, 24 (9.64%) were found to be good acid producers by changing the color of the medium completely from green to yellow within 48h. Of these 24 isolates, 11 (45.83%) were found to change the color within 12h of incubation and the remaining 13 (54.17%) LAB isolates completed the color change after 48h of incubation. Based on these results the isolates were examined for their acid production potential in relation to time of incubation.* Bacillus subtilis* and other unidentified bacterial species produced acid after 48h time of incubation. As indicated in Table 2 all the identified lactic acid bacteria (i.e.,* Lactobacillus paracasei, Enterococcus durans, Enterococcus hirae, Enterococcus avium, Lactobacillus brevis, *and* Enterococcus faecium*) were able to produce acid at 12 h time of incubation.

From 249 isolates 24 isolates were selected on the basis of their acid production potential. As Ashenafi [8], indicated, lactic acid bacteria were responsible for the acidic characteristics of the dough and reduced the pH to about 4.7 and were selected for molecular identification. Lactic acid bacteria produced acid at 12h of incubation. The color change is due to the production of lactic acid, and the pH was reduced so the color of bromophenol blue was changed from blue to yellow color.

Ayele et al. [22] have demonstrated that strong acid producing lactic acid bacteria belong to genera* Pediococcus, Lactobacillus, Streptococcus, Leuconostoc, *and* Bacillus* species. In addition, Brhanu, [23] has also showed that different genera of lactic acid bacteria were responsible for the acidic characteristics of the dough and these included* Pediococcus cerevisiae*,* Lactobacillus brevis*,* Lactobacillus plantarum, *and* Lactobacillus fermentum.*

### 3.3. Molecular Characterization of the Isolates

#### 3.3.1. Amplification and Sequencing of 16S rRNA Region of Bacterial Isolates

**Figure 2:**
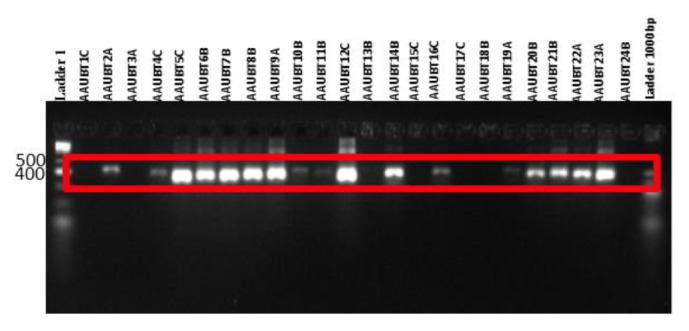
PCR amplification 16s rRNA using rD1 and fD1 bacterial universal primers.

All the isolates were shown to have PCR amplified fragments with around 500bp DNA. And the DNA of some isolates were not amplified; and no band was found; this may be due to the concentration sample DNA used for PCR amplification (Figure 2).

#### 3.3.2. 16S rRNA Sequence Analyses

After all the 16S rRNA sequences of 20 lactic acid bacteria were edited using the Bioedit software package, consensus sequences obtained were blasted in GenBank of NCBI and only 10 samples showed significant similarity of 92-98% in the GenBank. Accordingly, the 10 bacterial isolates were identified and belonging to genera* Lactobacillus* and* Enterococcus *and one genus* Bacillus *as presented in Table 3.

The two LAB genera contained six different species, namely,* Lactobacillus paracasei *(one isolate, AAUBT24B)*, Lactobacillus brevis *(one isolate, AAUBT21B)*, Enterococcus durans *(one isolate, AAUBT21B)*, Enterococcus hirae *(two isolates, AAUBT14B and AAUBT15C)*, Enterococcus avium *(two isolates, AAUBT13B and AAUBT19A), and* Enterococcus faecium *(one isolate, AAUBT12C). The genus* Bacillus* only was represented with one species and was named as* Bacillus subtilis *(two isolates AAUBT9A and AAUBT10B).

In this study* Lactobacillus paracasei, Enterococcus durans, Enterococcus hirae, Enterococcus avium, Lactobacillus brevis, Enterococcus faecium, and Bacillus subtilis* were also identified from fermenting teff dough. Different workers indicated that microbial flora of fermenting teff dough were complex and shown to include Enterobacteriaceae and aerobic mesophilic bacteria [8], aerobic spore formers, and lactic acid bacteria [7–9]. Brhanu et al. [9] have indicated that the microbes were involved in* teff* flour, indicating that there is the involvement of mold, Enterobacteriaceae, aerobic mesophilic bacteria, yeasts, fermentative aerogenic, Gram negative bacteria rods, lactic acid bacteria, and* Bacillus* spp.

*Bacillus subtilis* were also identified which is supported by Ayele et al. [22], who isolated and identified different* Bacillus* species from teff dough that included* Bacillus subtilis*,* Bacillus licheniformis, Bacillus circulans, Bacillus laterosporus, Bacillus firmus, Bacillus alvei, *and* Bacillus larvae*.

**Figure 3:**
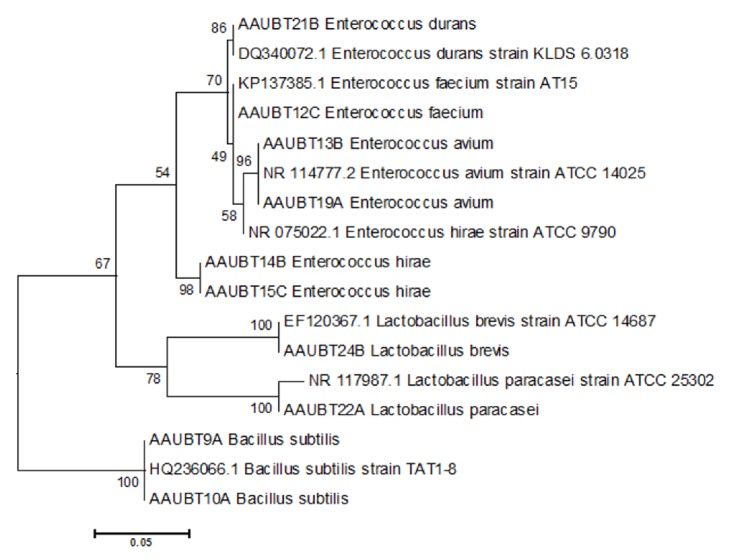
Phylogenetic tree of nucleotide sequence of 10 bacteria isolates from* teff* dough, i.e., AAUBT15C, AAUBT9A, AAUBT10A, AAUBT22A, AAUBT21B, AUBT24B, AAUBT13B, AAUBT1A9, AAUBT14B, and AAUBT12C. And 7 from GenBank were HQ236066.1, DQ340072.1, NR075022.1, NR117987.1, EF120367.1, NR 114777.2, and KP137385.1.

Phylogenetic tree made from sequenced 16S rRNA region of seven bacterial isolates of those identified from fermented* teff* dough (Table 3) and evolutionary analyses were conducted in MEGA6.06. The phylogenetic grouping indicated that strains having similar sequences were clustered in the same group and presumably were considered as close relatives. Maximum Likelihood phylogenetic trees based on the 16S rRNA genes of the isolated strains and their closest related species Bootstrap values calculated for 1000 replications are indicated, bar, 5 nt substitution per 100 nt indicated in Figure 3 with the sum of branch length of 0.567189932. The scale length of the tree was 0.05, with branch lengths in the same units as those of the evolutionary distances used to infer the phylogenetic tree. The evolutionary history was inferred by using the Maximum Likelihood method based on the Tamura-Nei model with the highest log likelihood value -1981.7596 and cutoff point 50. The tree was drawn to scale, with branch lengths measured in the number of substitutions per site. The analysis involved 24 nucleotide sequences. All positions containing gaps and missing data were eliminated. There were a total of 522 positions in the final dataset.

## 4. Conclusion

The study has indicated that different bacterial groups were involved in the fermentation process of* teff* dough. The result of this study may contribute to the future effort of the formulation of starter culture for* injera* dough fermentation.

## 5. Recommendation

It is important to examine the fermentation potential of each identified bacterial species in order to facilitate the formulation and development of starter cultures.

## Figures and Tables

**Table 1:** Characteristics of the colonial morphology of the isolated LAB and yeasts.

**Isolates **	**Species (16S rRNA gene analysis)**	**Colony Morphology**				
		**Pigmentation**	** Shape **	**Elevation **	**Size **	**Gram stain**
AAUBT1C	Unidentified	White	Circular	Flat	Small	+
AAUBT3A	Unidentified	White	Circular	Flat	Small	+
AAUBT4C	Unidentified	White	Circular	Flat	Small	+
AAUBT5C	Unidentified	White	Circular	Flat	Small	+
AAUBT6B	Unidentified	White	Circular	Flat	Small	+
AAUBT8B	Unidentified	White	Circular	Flat	Small	+
AAUBT9A	*Bacillus subtilis*	White	Circular	Flat	Small	+
AAUBT10B	*Bacillus subtilis*	White	Irregular	Flat	Small	+
AAUBT11B	Unidentified	White	Circular	Flat	Small	+
AAUBT12C	*Enterococcus faecium*	White	Circular	Flat	Small	+
AAUBT13B	*Enterococcus avium *	White	Circular	Flat	Small	+
AAUBT14B	*Enterococcus hirae *	White	Circular	Flat	Small	+
AAUBT15C	*Enterococcus hirae *	White	Circular	Flat	Small	+
AAUBT16C	Unidentified	White	Irregular	Flat	Small	+
AAUBT18B	Unidentified	White	Circular	Flat	Small	+
AAUBT19A	*Enterococcus avium *	White	Irregular	Flat	Small	+
AAUBT21B	*Enterococcus durans*	White	Circular	Flat	Small	+
AAUBT22A	*Lactobacillus paracasei *	White	Circular	Flat	Small	+
AAUBT23A	Unidentified	White	Circular	Flat	Small	+
AAUBT24B	*Lactobacillus brevis *	White	Irregular	Flat	Small	+

**Table 2:** Acid production capacity of the isolates after incubation.

Species	Frequency of bacterial isolate (no./%)		Total
	12h	48h	
*Bacillus subtilis*	-	2(10%)	2(10%)
*Enterococcus avium*	2(10%)	-	2(10%)
*Enterococcus durans*	1(5%)	-	1(5%)
*Enterococcus faecium*	1(5%)	-	1(5%)
*Enterococcus hirae*	2(10%)	-	2(10%)
*Lactobacillus brevis*	1(5%)	-	1(5%)
*Lactobacillus paracasei*	1(5%)	-	1(5%)
Unidentified LAB	2(10%)	8(40%)	10(50%)
Total	10(50%)	10(50%)	20(100%)

**Table 3:** Phylogenetic neighbors of bacteria on the basis of similarity to the partial 16S rDNA sequence.

**Sequence ID**	**E-value**	**Identity**	**Species (16S rRNA gene analysis)**	**Accession**
AAUBT9A	0.0	98%	*Bacillus subtilis *TAT1-8	HQ_236066.1
AAUBT10B	0.0	98%	*Bacillus subtilis *TAT1-8	HQ_236066.1
AAUBT19A	0.0	94%	*Enterococcus avium *ATCC 14025	NR_114777.2
AAUBT13B	0.0	92%	*Enterococcus avium *ATCC 14025	NR_114777.2
AAUBT21B	0.0	97%	*Enterococcus durans KLDS* 6.0318	DQ_340072.1
AAUBT12C	0.0	98%	*Enterococcus faecium *AT15	KP_137385.1
AAUBT15C	0.0	94%	*Enterococcus hirae *ATCC 9790	NR_075022.1
AAUBT14B	0.0	97%	*Enterococcus hirae *ATCC 9790	NR_075022.1
AAUBT24B	0.0	92%	*Lactobacillus brevis *ATCC 14687	EF_120367.1
AAUBT22A	0.0	97%	*Lactobacillus paracasei *ATCC 25302	NR_117987.1

## Data Availability

The data that support the findings of this study are available from the corresponding author upon sensible request.
